# Baicalein Attenuates Pyroptosis and Endoplasmic Reticulum Stress Following Spinal Cord Ischemia-Reperfusion Injury *via* Autophagy Enhancement

**DOI:** 10.3389/fphar.2020.01076

**Published:** 2020-07-30

**Authors:** Chenyu Wu, Hui Xu, Jiafeng Li, Xinli Hu, Xingyu Wang, Yijia Huang, Yao Li, Sunren Sheng, Yongli Wang, Huazi Xu, Wenfei Ni, Kailiang Zhou

**Affiliations:** ^1^ Department of Orthopaedics, The Second Affiliated Hospital and Yuying Children’s Hospital of Wenzhou Medical University, Wenzhou, China; ^2^ Zhejiang Provincial Key Laboratory of Orthopaedics, Wenzhou, China; ^3^ Department of Orthopaedics, Huzhou Central Hospital, Huzhou, China

**Keywords:** baicalein, spinal cord ischemia-reperfusion injury, autophagy, pyroptosis, endoplasmic reticulum stress

## Abstract

**Background:**

Spinal cord ischemia-reperfusion injury (SCIRI) is the main complication after the repair of a complex thoracoabdominal aortic aneurysm. Many clinical treatments are not ideal due to the complex pathophysiological process of this injury. Baicalein (BA), a component derived from the roots of the herb *Scutellaria baicalensis*, may contribute to the successful treatment of ischemia/reperfusion injury.

**Purpose:**

In the present study, the effects of BA on spinal cord ischemia-reperfusion injury and the underlying mechanisms were assessed.

**Materials and Methods:**

Spinal cord ischemia was induced in C57BL/6 mice by blocking the aortic arch. Fifty-five mice were then randomly divided into four groups: Sham, SCIR+Vehicle, SCIR+BA, and SCIR+BA +3MA groups. At 0 and 24 h pre-SCIRI and at 24 h and 7 days post-SCIRI, evaluations with the Basso mouse scale (BMS) were performed. On postoperative 24 h, the spinal cord was harvested to assess pyroptosis, endoplasmic reticulum stress mediated apoptosis and autophagy.

**Results:**

BA enhanced the functional recovery of spinal cord ischemia-reperfusion injury. In addition, BA attenuated pyroptosis, alleviated endoplasmic reticulum stress-mediated apoptosis, and activated autophagy. However, the effects of BA on the functional recovery of SCIRI, pyroptosis and endoplasmic reticulum stress-mediated apoptosis were reversed by the inhibition of autophagy.

**Conclusions:**

In general, our findings revealed that BA enhances the functional recovery of spinal cord ischemia-reperfusion injury by dampening pyroptosis and alleviating endoplasmic reticulum stress-mediated apoptosis, which are mediated by the activation of autophagy.

## Introduction

Spinal cord ischemia-reperfusion injury (SCIRI) is the main complication after the repair of a complex thoracoabdominal aortic aneurysm, which secondarily causes nerve injury and thereby leads to deficits in movement and sensation, as well as a wide range of disabilities (Miranda et al., 2018). SCIRI, with an incidence of approximately 5% to 10%, seriously affects patients’ quality of life (Conrad et al., 2008). Many treatment measures have been used to cure SCIRI. These include preconditioning treatments (such as remote ischemic preconditioning (Karimipour et al., 2019) and hyperbaric oxygen preconditioning (Hentia et al., 2018), pharmacologic interventions [dexmedetomidine (Ha Sen Ta et al., 2019), minocycline and progesterone (Faheem et al., 2019)] and other clinical management strategies including the use of pluripotent stem cells and hypothermia (Jia et al., 2013). However, the functional recovery of most patients after SCIRI is not ideal. Therefore, it is necessary to explore the pathological process of SCIRI to find a new treatment. In SCIRI, nerve cells are vulnerable to ischemic injury due to their high demand for energy, and the death of nerve cells is an important reason for their slow recovery of patients (Li et al., 2018a). At present, according to previous reports, there are three common death modes of neurons in SCIRI: pyroptosis, apoptosis and autophagy (Wang et al., 2017; Chen et al., 2019; Dai et al., 2019).

Pyroptosis is a programmed inflammatory necrotizing cell death mediated by gasdermin D (GSDMD) (Shi et al., 2017). Its main characteristic is the activation of the inflammasome, represented by NLRP3, which can recruit and activate the proteolytic enzyme caspase-1 leading to the cleavage of the pore-forming protein GSDMD and the maturation of the proinflammatory cytokines interleukin-1β (IL-1β) and IL18 (Zhang et al., 2019). Studies have indicated that the production of reactive oxygen species (ROS) can activate the NLRP3 inflammasome (Qiu et al., 2019). It has been indicated that SCIRI can induce nerve cell pyroptosis, leading to neurological impairment (Xia et al., 2018). Moreover, under the conditions of SCIRI, the unfolded protein response (UPR) of the endoplasmic reticulum (ER) is induced to react. In addition, persistent and serious ER-stress (ERS) activates the ERS related apoptosis pathway, which leads to apoptosis (Zhao et al., 2019). In spinal cord injury, ERS mediated apoptosis will lead to the aggravation of demyelination, the destruction of the blood-spinal cord barrier (BSCB) and disorders of motor recovery (Chen et al., 2019). In recent years, a growing number of studies have focused on the treatment of SCIRI by reducing ER stress-induced SCIRI (Mizukami et al., 2010; Wu et al., 2018).

Autophagy, an evolutionarily conserved catabolic process, facilitates the recycling of damaged cellular proteins and organelles. It is widely accepted that autophagy is a “double-edged sword.” On one hand, autophagy can induce autophagic cell death and accelerate cell death; on the other hand, autophagy can promote the circulation of protein and organelles in damaged cells, and is an essential homeostasis process for cells to decompose their own components (Rathinam et al., 2012). In a study of SCIRI, autophagy was considered to play an important role in SCIRI, as Wang et al. have reported that the induction of autophagy can protect motoneurons from degeneration and alleviate the spastic paralysis following SCIRI (Wang et al., 2017). Recently, an increasing number of studies have found that the activation of autophagy can inhibit pyroptosis. Previous studies have reported that mitophagy could reduce mitochondrial ROS and subsequent NLRP3 inflammasome activation in kidney injury (Lin et al., 2019b). Autophagy can not only inhibit the activation of NLRP3 inflammasomes, but also capture and reduce pro-IL-1β and IL-18, limiting their secretion and inhibiting pyroptosis (Byrne et al., 2013). In addition, it has been shown that autophagy can partially reduce ERS-mediated apoptosis by eliminating the aggregation of unfolded proteins (Li et al., 2019). Therefore, autophagy is a promising target to depress pyroptosis and ERS mediated apoptosis for the treatment of SCIRI.

Baicalein (5,6,7-trihydroxyflavone, a flavone subclass of flavonoids), is one of the major components of the roots of the herb *Scutellaria baicalensis* (Li et al., 2018b). Due to its multiple biological activities, baicalein has successfully been used in the treatment of cardiac injury, nerve injury and liver injury (Chang et al., 2011; Zhang et al., 2012; Liu et al., 2015). Recent studies have shown that baicalein can induce autophagy to prevent hepatocellular injury (Liu et al., 2016). It can block NLRP3-GSDMD signaling to reduce pyroptosis and thereby protect the liver (Shi et al., 2020). In addition, baicalein can inhibit CHOP expression, suggesting that BA can suppress ER stress-induced apoptosis by inhibiting the CHOP pathway (Choi et al., 2010). However, whether baicalein has a therapeutic effect on SCIRI is not known. In this study, the effect of baicalein on the functional recovery of SCIRI was investigated in the mouse. Through histological and protein analyses, the underlying mechanisms of baicalein in autophagy activation to inhibit pyroptosis and ER stress-induced apoptosis were analyzed in an SCIRI model.

## Materials and Methods

### Ethical Statement

All animals were maintained in accordance with the Guide for the Care and Use of Laboratory Animals of the China National Institutes of Health. The animals used in this study were approved by the Animal Research Committee of Wenzhou Medical University (wydw2017-0022).

### Reagents

Baicalein (C_15_H_10_O_5_, HPLC purity 98%) was purchased from Tauto Biotechnology (Shanghai, China). Pentobarbital sodium was provided by Solarbio Science & Technology (Beijing, China). A rabbit monoclonal anti-GAPDH antibody was acquired from Biogot Technology (AP0063; Shanghai, China). Primary antibodies against NLRP3 (Cat. No. 15101), Beclin1 (Cat. No. 3738), ATG5 (Cat. No. 12994), ATP6V1B2 (Cat. No. 14617), ATF4 (Cat. No. 11815), and CHOP (Cat. No. 2985) were purchased from Cell Signaling Technology (Beverly, MA, USA). The rabbit monoclonal anti-phosphoinositide-3-kinase (VPS34) and anti-cathepsin D (CTSD) antibodies were acquired from the Proteintech Group (12452-1 and 21327-1; Chicago, IL, USA). The mouse monoclonal anti-SQSTM1/-p62 antibody was purchased from Abcam (ab56416; Cambridge, UK). Rabbit monoclonal anti-microtubule-associated 1 protein light chain 3 (LC3) antibody and 3MA were purchased from Sigma-Aldrich Chemical Co. (L7543 and M9281; Milwaukee, WI, USA). Antibodies against GRP78 (Cat. No. ab21685), PDI (Cat. No. ab2792), CASP12 (Cat. No. ab62484) and NeuN (Cat. No. ab177487) were obtained from Abcam (Cambridge, UK). Antibodies against GSDMD (Cat. No. af4013), C-CASP1 (Cat. No. af4022) were purchased from Affinity Biosciences (OH.USA). Horseradish peroxidase (HRP)-conjugated immunoglobulin G (IgG) secondary antibody was provided by Santa Cruz Biotechnology (Dallas, TX, USA). Fluorescein isothiocyanate (FITC)-conjugated IgG secondary antibody was obtained from Boyun Biotechnology (Nanjing, China), and the 4′,6-diamidino- 2-phenylindole (DAPI) solution was purchased from Beyotime Biotechnology (Jiangsu, China). An Electrochemiluminescence (ECL) Plus Reagent Kit was obtained from PerkinElmer Life Sciences (Waltham, MA, USA), and a BCA Kit was acquired from ThermoFisher Scientific (Rockford, IL, USA).

### Animals and Groups

**Figure 1:**
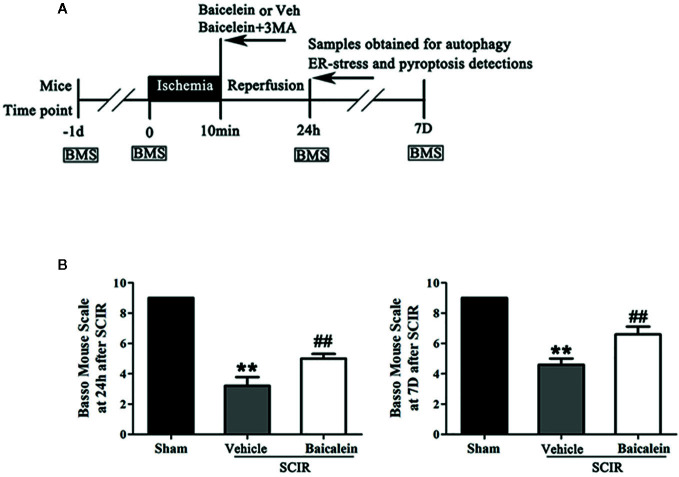
Baicalein promotes functional recovery after spinal cord ischemia-reperfusion injury (SCIR). **(A)** Schematic diagram of the experimental design. Each group received vehicle, baicalein (100 mg/kg, *ip*), or baicalein plus 3-methyladenine (3MA) (15 mg/kg, *ip*). Mice received these treatments at the beginning of reperfusion and were sacrificed at 24 h and 7 days after SCIR. **(B)** BMS scores of mice in the Sham, SCIR+Vehicle, and SCIR+Baicalein groups at 24 h and 7 days after SCIR. The values are expressed as the means ± SEM, n = 5 per group. **p < 0.01, vs. Sham group. ^##^p < 0.01, vs. SCIR+Vehicle group.

C57BL/6 mice (male, 20–30 g) were purchased from Wenzhou Medical University’s Experimental Animal Center (license no. SCXK [ZJ] 2005-0019), Zhejiang Province, China and were allowed to adapt to their new location for a week. The animals were maintained individually in experimental cages with a 12-h light-dark natural cycle and free access to a standard diet and water. Fifty-five mice were randomly divided into four groups: Sham (n = 15), SCIR+Vehicle (n = 15), SCIR+Baicalein (SCI+BA, n = 15), and SCIR+BA+3-methyladenine (SCI+BA +3MA, n = 10). A schematic diagram of the experimental design is shown in **Figure 1A**. All the experimental procedures were carried out in accordance with the Guide for the Care and Use of Laboratory Animals of the China National Institutes of Health.

### SCIRI Model

The model of SCIRI was carried out according to a previous study (Li et al., 2014). All the mice were anesthetized using 1% (w/v) pentobarbital sodium (50 mg/kg) *via* intraperitoneal injection and placed in the supine position. Ischemia of the spinal cord was produced by blocking the aortic arch for 10 min. In the sham group, the aortic arch was exposed with no blocking.

### Drug Administration

Baicalein was dissolved in a solution of 2% DMSO in normal saline. Daily intraperitoneal injection of 100 mg/kg BA was performed for the SCIR+BA group. The SCIR+ BA + 3MA group was treated with 3MA (15 mg/kg) 30 min before each BA administration. All drugs were injected for seven consecutive days, from before the operation until death.

### Basso Mouse Scale

The Basso mouse scale (BMS) was used to evaluate the functional consequences of SCIRI as described previously (Basso et al., 2006). In brief, the BMS ranges from a score of 0 for complete paraplegia to a score of 9 for normal function. The test was carried out in an open field and the BMS scores were measured at 0 and 24 h pre-SCIRI and at 24 h and 7 days post-SCIRI by two investigators who were blinded to the experimental conditions.

### Immunofluorescence Staining

After the euthanasia of the mice on postoperative day 1, the spinal cord segments at L_2-3_ were collected and fixed in 4% (w/v) paraformaldehyde for 24 h. The samples were then embedded in paraffin for transverse paraffin sections. The paraffin sections (4-μm thick) were mounted on poly-L-lysine-coated slides for immunofluorescence staining. Next, the sections were deparaffinized, rehydrated, washed, and then treated with 10.2 mM sodium citrate buffer for 10 min at 95°C. The sections were permeabilized with 0.1% (v/v) PBS-Triton X-100 for 30 min. After blocking with 10% (v/v) bovine serum albumin in PBS for 30 min, the samples were incubated with antibodies against LC3 (1:200)/NeuN (1:400), p62 (1:200)/NeuN (1:400), and CASP12/NeuN, C-CASP1 (1:300)/NeuN (1:400) at 4°C overnight. After this, the specimens were incubated at room temperature for 1 h with secondary antibodies. A fluorescence microscope (Olympus, Tokyo, Japan) was used to visualize and evaluate the spinal cord in six randomly selected fields from three random sections of each sample.

### Western Blotting

After the mice were euthanized 24 h after the SCIRI, the spinal cord tissue was dissected and stored at −80°C for Western blotting. Total proteins from the spinal cord tissues were purified using protein extraction reagents and the proteins were measured using the BCA assay. Equal amounts of protein (60 μg) per mouse were separated by 12% (w/v) gel electrophoresis and transferred to polyvinylidene fluoride (PVDF) membranes (Roche Applied Science, Indianapolis, IN, USA). After blocking with 5% (w/v) nonfat milk for 2 h at room temperature, the membranes were incubated with the subsequent primary antibodies at 4°C overnight: ATG5(1:1000), ATF4(1:1,000), ATP6V1B2 (1:1,000), CHOP (1:1,000), PDI (1:1,000), NLRP3 (1:1,000), GSDMD (1:1,000), CASP12(1:1,000), GRP78 (1:1,000), ASC (1:1,000), C-CAPS1 (1:1,000), Beclin1 (1:1,000), p62 (1:1,000), LC3 (1:500), VPS34 (1:1,000), CTSD (1:1,000), and GAPDH (1:1,000). The membranes were then incubated with HRP-conjugated IgG secondary antibody (1:5,000) for 2 h at room temperature. The bands on the membranes were visualized using an ECL Plus Reagent Kit. Finally, the band intensity was quantified using Image Lab 3.0 software (Bio-Rad, Hercules, CA, USA).

### Terminal Deoxynucleotidyl Transferase dUTP Nick End Labeling Assay

Transferase dUTP nick end labeling (TUNEL) was carried out to detect the DNA fragmentation due to the apoptotic signaling cascades. The slides with the spinal cord tissue were incubated with proteinase K for 30 min at 37°C, after which they were processed with an In Situ Cell Death Detection Kit according to the manufacturer’s instructions Images were visualized with a fluorescence microscope (Olympus, Tokyo, Japan) at a ×200 magnification. The TUNEL positive cells were automatically counted in six randomly selected fields from each slide per sample by using the IPP software.

### Enzyme-Linked Immunosorbent Assay

The BMS of mice was used to evaluate locomotor function recovery, in the Sham, SCIR+Vehicle, and SCIR+Baicalein groups at 24 h and 7 days after SCIR. In the Sham group, the BMS scores were significant higher than those in the SCIR+Vehicle group at 24 h and 7 day after SCIR (respectively, 9 vs. 3.2 ± 1.3; 9 vs. 4.6 ± 0.89; *P* < 0.001, *P* < 0.001); after treatment with baicalein, the SCIR group obtained higher BMS scores at 24 h and 7 days after SCIR (5.2 ± 0.8 and 6.6 ± 1.1 respectively) than the SCIR+Vehicle group (*P* = 0.004, *P* = 0.003, respectively; **Figure 1B**).

### Baicalein Enhances Autophagy After SCIR

**Figure 2:**
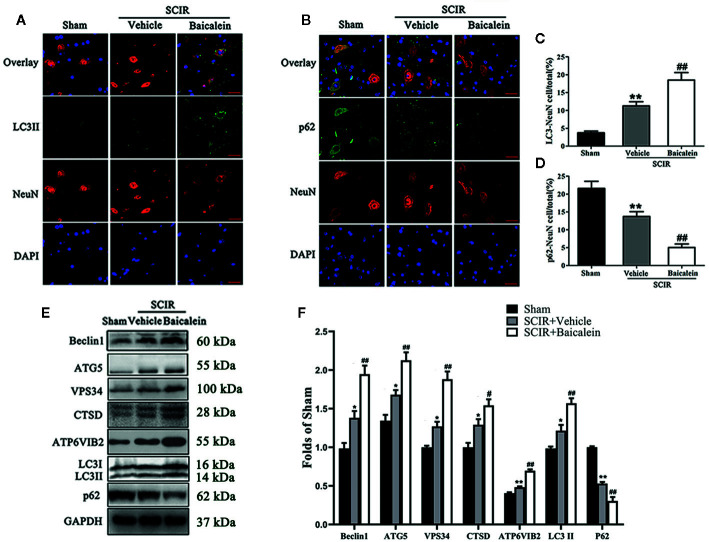
Baicalein enhances autophagy after spinal cord ischemia-reperfusion injury (SCIR). **(A)** Immunofluorescence staining for LC3II and NeuN colocalization at the spinal cord lesion after SCIR (Scan bar = 25μm). **(B)** Immunofluorescence staining for p62 and NeuN colocalization at the spinal cord lesion after SCIR (Scan bar = 25μm). **(C)** The quantitative percentage of the LC3II positive neurons at the spinal cord lesion in each group. **(D)** The quantitative percentage of the p62 positive neurons in each group. **(E)** Western blotting for Beclin1, ATG5, VPS34, CTSD, ATP6V1B2, LC3II, and p62 expression levels in the Sham, SCIR+Vehicle, and SCIR+Baicalein groups. The gels were run under the same experimental conditions, and the cropped blots are shown here. **(F)** The optical density values of the Beclin1, ATG5, VPS34, CTSD, ATP6V1B2, LC3II, and p62 expression levels were quantified and analyzed in each group. The values are expressed as the means ± SEM, n=5 per group. *p < 0.05 and **p < 0.01, vs. Sham group. ^##^ *p* < 0.01, vs. SCIR+Vehicle group.

To assess the level of autophagy at the spinal cord lesion after SCIR, we tested the autophagosomal markers Beclin1, ATG5, VPS34, and LC3II, as well as autolysosome-related markers CTSD and ATP6V1B2, and the substrate protein p62. As shown in **Figure 2A**, the autophagosomes were labeled with LC3II punctate dots (green), the neurons were labeled with NeuN (red), and the nuclei were labeled with DAPI (blue) at the spinal cord. The spinal cord exhibited a higher percentage of LC3II-positive neurons (11.3 ± 2.6%) in the SCIR+Vehicle group than in the Sham group(3.3 ± 0.5%; *P* = 0.002); baicalein treatment further increased the percentage of LC3II-positive neurons in the SCIR mice (18.5 ± 2.1%; *P* = 0.003; **Figure 2C**). As shown in **Figure 2B**, immunofluorescence staining was also performed to show p62 expression in the neurons at the lesion; p62 was labeled in green, the neurons were labeled in red, and the nuclei were labeled in blue. After quantitative analysis, the results showed that the percentage of p62-positive neurons significantly decreased after SCIR (13.7 ± 2.9% vs. 21.6 ± 4.7%; *P* =0.003); moreover, a lower percentage of p62-positive neurons was observed in the SCIR+Baicalein group (5.0 ± 2.2%), compared with the SCIR+Vehicle group (*P* = 0.001; **Figure 2D**). The expression levels of Beclin1, ATG5, VPS34, CTSD, ATP6V1B2, LC3II and p62 were detected by Western blotting (**Figure 2E**). The results showed that the optical density (OD) values for Beclin1, ATG5, VPS34, CTSD, ATP6V1B2 and LC3II were higher in the SCIR+Vehicle group than the Sham group (*P* = 0.011, *P* = 0.014, *P* = 0.017, *P* = 0.012, *P* = 0.002, *P* = 0.018, respectively), with lower OD value of p62 in the SCIR+Vehicle group (*P* < 0.001), while baicalein further enhanced the alterations of these markers’ OD values in the mice following SCIR (*P* = 0.001, *P* = 0.002, *P* < 0.001, *P* = 0.026, *P* < 0.001, *P* = 0.001, *P* < 0.001, respectively; **Figure 2F**).

### Baicalein Inhibits ER Stress After SCIR

**Figure 3:**
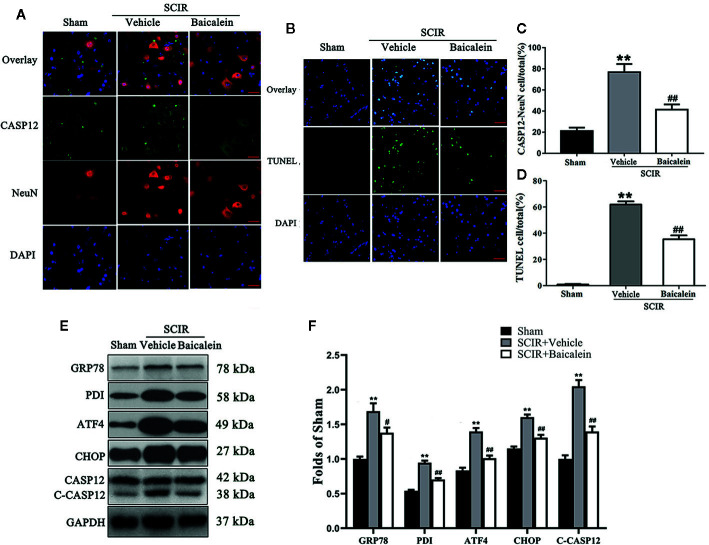
Baicalein inhibits endoplasmic reticulum (ER) stress after spinal cord ischemia-reperfusion injury (SCIR). **(A)** Immunofluorescence staining for CASP12 and NeuN colocalization in the spinal cords of the Sham, SCIR+Vehicle, and SCIR+Baicalein groups (Scan bar= 25μm). **(B)** Transferase dUTP nick end labeling (TUNEL) staining for labeling apoptotic cells at the spinal cord lesion after SCIR. **(C)** The quantitative percentage of the CASP12-positive neurons in the Sham, SCIR+Vehicle, and SCIR+Baicalein groups. **(D)** The quantitative percentage of the TUNEL-positive cells at the spinal cord lesion in each group. **(E)** Western blotting for GRP78, PDI, ATF4, CHOP, and CASP12 expression levels in the Sham, SCIR+Vehicle, and SCIR+Baicalein groups. The gels were run under the same experimental conditions, and the cropped blots are shown here. **(F)** The optical density values of the GRP78, PDI, ATF4, CHOP, and CASP12 expression levels were quantified and analyzed in each group. The values are expressed as the means ± SEM, n=5 per group. **p < 0.01, vs. Sham group. ^#^ *p* < 0.05 and ^##^ *p* < 0.01, vs. SCIR+Vehicle group.

To assess the status of ER stress and whether it induced apoptosis in SCIR, we carried out the evaluation of ER stress-related proteins GRP78, PDI, ATF4, CHOP and CASP12, and TUNEL staining. As shown in **Figure 3A**, immunofluorescence staining was performed to show the expression of CASP12 in neurons at the lesion; CASP12 was labeled in green, the neurons were labeled in red, and the nuclei were labeled in blue. After quantitative analysis, the results showed that the percentage of CASP12-positive neurons significantly increased at the spinal cord after SCIR (76.8 ± 7.8% vs. 21.1 ± 3.3%; *P* < 0.001); however baicalein decreased the percentage of CASP12-positive neurons at the spinal cord lesion (41.4 ± 5.0%; *P* < 0.001; **Figure 3C**). TUNEL staining was carried out to examine the apoptosis levels *via* DNA fragmentation under conditions of ER stress. As shown in **Figure 3B**, the apoptotic cells were labeled in green, and the nuclei were labeled in blue. Quantitative analysis indicated that the percentage of apoptotic cells in the SCIR+Vehicle group (61.9 ± 5.3%) was significantly higher than that in Sham group (0.8 ± 0.4%; *P* < 0.001); baicalein decreased the percentage of apoptotic cells at the spinal cord lesion (35.4 ± 6.5%; *P* < 0.001; **Figure 3D**). The expression levels of GRP78, PDI, ATF4, CHOP and CASP12 were detected by Western blotting (**Figure 3E**). The results revealed that the OD values for GRP78, PDI, ATF4, CHOP, and CASP12 were higher in the SCIR+Vehicle group than in the Sham group (*P* < 0.001, *P* < 0.001, *P* < 0.001, *P* < 0.001, *P* < 0.001, respectively); however, decreases in the OD values for GRP78, PDI, ATF4, CHOP and CASP12 were detected in the SCRI+Baicalein group relative to the SCRI+Vehicle group (*P* = 0.018, *P* < 0.001, *P* < 0.001, *P* < 0.001, *P* < 0.001, *P* < 0.001, respectively; **Figure 3F**).

### Baicalein Attenuates Pyroptosis After SCIR

**Figure 4:**
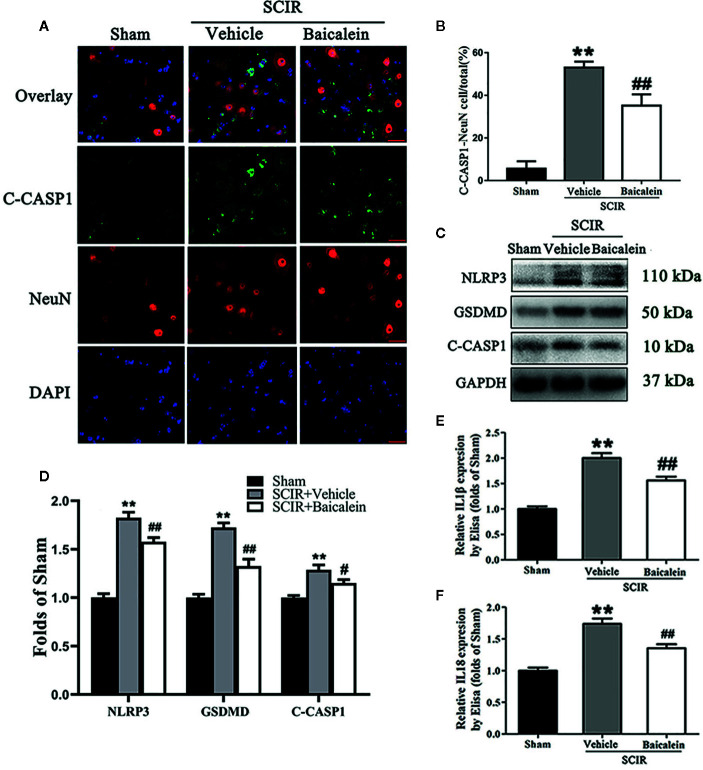
Baicalein attenuates pyroptosis after spinal cord ischemia-reperfusion injury (SCIR). **(A)** Immunofluorescence staining for C-CASP1 and NeuN colocalization in the spinal cords of the Sham, SCIR+Vehicle, and SCIR+Baicalein groups (Scan bar= 25μm) **(B)** The quantitative percentage of the C-CASP1-positive neurons at the spinal cord lesion. **(C)** Western blotting for NLRP3, GSDMD, and C-CASP1 expression levels in the Sham, SCIR+Vehicle, and SCIR+Baicalein groups. The gels were run under the same experimental conditions, and the cropped blots are shown here. **(D)** The optical density values of the NLRP3, GSDMD, and C-CASP1 expression levels were quantified and analyzed in each group. **(E, F)** Enzyme-linked immunosorbent assay (ELISA) results for IL1βand IL18 expression levels in the Sham, SCIR+Vehicle, and SCIR+Baicalein groups. The values are expressed as the means ± SEM, n=5 per group. **p < 0.01, vs. Sham group. ^#^ *p* < 0.05 and ^##^ *p* < 0.01, vs. SCIR+Vehicle group.

Biomarkers NLRP3, GSDMD, C-CASP1, IL1β, and IL18 were assessed in the spinal cord after SCIR to determine the pyroptosis level in the Sham, SCIR+Vehicle and SCIR+Baicalein groups. Colocalization of C-CASP1 (green) and NeuN (red) at the spinal cord lesion by immunofluorescence staining was performed for each group (**Figure 4A** **)**. As shown in **Figure 4B**, the percentage of the pyroptotic neurons was significantly increased in the spinal cord lesions in the SCIR+Vehicle group relative to the Sham group (respectively, 53.0 ± 6.8 vs. 5.6 ± 8.6%; *P* < 0.001), while baicalein decreased the percentage of pyroptotic neurons at the spinal cord with SCIR (35.2 ± 13.0%; *P* = 0.007). Western blotting for NLRP3, GSDMD and C-CASP1 expressions levels was carried out for each group (**Figure 4C**). The results showed that the OD values for NLRP3, GSDMD and C-CASP1 were higher in the SCIR+Vehicle group than in the Sham group (*P* < 0.001, *P <*0.001, *P* < 0.001, respectively); however, decreases in the OD values for these markers were detected in the SCRI+Baicalein group relative to the SCRI+Vehicle group (*P* = 0.003, *P* < 0.001, *P* = 0.027, respectively; **Figure 4D**). The levels of IL1β and IL18 were detected by ELISA, and the results revealed that both IL1β and IL18 level in the SCIR+Vehicle group were increased, compared with those in the Sham group (*P* < 0.001, *P <*0.001, respectively), while baicalein reversed the alterations of IL1β and IL18 level in the mice following SCIR (*P* < 0.001, *P* = 0.001, respectively; **Figures 4E, F** **)**.

### Inhibition of Autophagy Reverses the Effects of Baicalein on ER Stress, Pyroptosis, and Functional Recovery After SCIR

**Figure 5:**
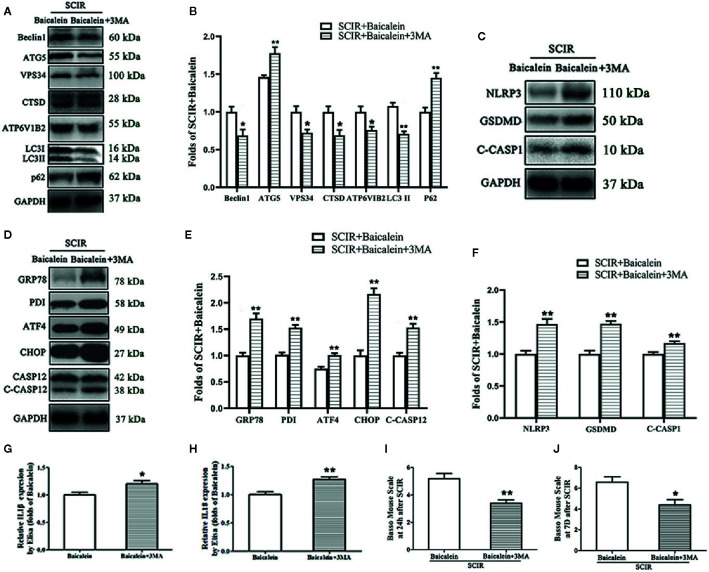
Inhibition of autophagy reverses the effects of baicalein on endoplasmic reticulum (ER) stress, pyroptosis, and functional recovery after SCIR. **(A)** Western blotting for the Beclin1, ATG5, VPS34, CTSD, ATP6V1B2, LC3II and p62 expression levels in the SCIR+Baicalein, and SCIR+3MA groups. The gels were run under the same experimental conditions, and the cropped blots are shown here. **(B)** The optical density values of the Beclin1, ATG5, VPS34, CTSD, ATP6V1B2, LC3II and p62 expression levels were quantified and analyzed in each group. **(C, D)** Western blotting for the NLRP3, GSDMD, C-CASP1, GRP78, PDI, ATF4, CHOP, and CASP12 expression levels in the SCIR+Baicalein and SCIR+3MA groups. The gels were run under the same experimental conditions, and the cropped blots are shown here. **(E, F)** The optical density values of GRP78, PDI, ATF4, CHOP, CASP12, NLRP3, GSDM, and C-CASP1expression levels were quantified and analyzed in the both groups. **(G, H)** Enzyme-linked immunosorbent assay (ELISA) results for IL1βand IL18 expression levels in the SCIR+Baicalein and SCIR+3MA groups. **(I, J)** The BMS scores of the mice in each group at 24 h and 7 days after SCIR. The values are expressed as the means ± SEM, n=5 per group. ^*^ *p* < 0.05 and ^**^ *p* < 0.01, vs. SCIR+Baicalein group.

The levels of autophagy, ER stress and pyroptosis in SCIR after autophagy inhibition were evaluated by Western blotting. The expression levels of Beclin1, ATG5, VPS34, CTSD, ATP6V1B2, LC3II, and p62 were detected by Western blotting (**Figure 5A**). The results showed that the OD values for Beclin1, ATG5, VPS34, CTSD, ATP6V1B2, and LC3II were lower in the SCIR+Baicalein+3MA group than in the SCIR+Baicalein group (*P* = 0.017, *P* = 0.006, *P* = 0.013, *P* = 0.017, *P* = 0.025, *P* < 0.001, respectively), with a higher OD value for p62 in the SCIR+Baicalein group (*P* = 0.001; **Figure 5B**). The expression levels of NLRP3, GSDMD, C-CASP1, GRP78, PDI, ATF4, CHOP, and CASP12 were also tested by Western blotting (**Figures 5C, D** **)**. The results revealed that the OD values for GRP78, PDI, ATF4, CHOP, CASP12, NLRP3, GSDMD, and C-CASP1 were higher in the SCIR+Baicalein+3MA group than in the SCIR+Baicalein group (*P* < 0.001, *P* < 0.001, *P* = 0.001, *P* < 0.001, *P* < 0.001, *P* =0.001, *P* < 0.001, *P* = 0.004 respectively; **Figures 5E, F** **)**. The levels of IL1β and IL18 were detected by ELISA, and the results showed that both IL1β and IL18 levels in the SCIR+Baicalein+3MA group were increased, compared with those in the SCIR+Baicalein group (*P* = 0.031, *P* = 0.003, respectively; **Figures 5G, H** **)**. In the SCIR+Baicalein+3MA group, the BMS scores were significantly lower than those in the SCIR+Baicalein group at 24 h and 7 days after SCIR (respectively, 5.2 ± 0.84 vs. 3.4 ± 0.55; 6.6 ± 1.14 vs. 4.4 ± 1.14; *P* = 0.004, *P* = 0.016; **Figures 5I, J** **)**.

## Discussion

Baicalein is a naturally occurring bioflavonoid, that has gained considerable attention in various fields, owing to its diverse biological activities (Li et al., 2018b). Many studies have demonstrated that it plays a significant role in the treatment of various diseases, including cerebral injury, Parkinson’s disease (PD), cardiac injury and liver injury, by promoting autophagy and inhibiting oxidative stress, inflammation and cell apoptosis (Chang et al., 2011; Li et al., 2018b). SCIRI, which is a severe complication after thoracoabdominal aortic surgery, causes a wide range of complication. Whether BA is able to treat SCIRI remains unknown. Our study revealed that BA played a part in the treatment of SCIRI and that the underlying mechanism involves promoting autophagy to dampen pyroptosis and ERS mediated apoptosis.

Baicalein reportedly attenuates hepatic injury in nonalcoholic steatohepatitis by suppressing pyroptosis (Shi et al., 2020). Pyroptosis, a specific pro-inflammatory programmed cell death, is characterized by the swelling and rupture of cells and the lysis and release of pro-inflammatory molecules (Xing et al., 2020; Zheng and Li, 2020). Pyroptosis is considered an important factor in models of renal, hepatic and myocardial ischemia and reperfusion injury (Wu et al., 2016; Qiu et al., 2017; Zhang et al., 2019). Consequently, we speculated that BA may downregulate pyroptosis in SCIRI. We examined the level of pyroptosis in the spinal cord tissue. The hypoxic/ischemic microenvironment activates inflammasomes, as NLRP3 is recruited to induce the activation of caspase1. Caspase-1 activates the inflammatory factors IL-1 β and IL-18 (Cheng et al., 2019). Caspase-1 cleaves GSDMD to release active GSDMD-N, which is transferred to the plasma membrane to cause pyroptosis (Zheng and Li, 2020). Therefore, we examined NLRP3, GSDMD, Caspase1, IL1βand IL18 expression levels to determine the extent of pyroptosis in SCIRI. ELISA revealed that BA apparently inhibited the levels of IL1β and IL18, while, Western blotting results showed that BA obviously depressed the levels of NLRP3, GSDMD and C-CASP1. Moreover, immunofluorescence staining for NeuN/C-CASP1 revealed that BA decreased the C-CASP1-positive neurons following SCIRI. These results indicated that BA attenuated pyroptosis in SCIRI.

ER stress refers to the accumulation of misfolded proteins in the organelle, which is caused by genetic or external factors that hinder the ability of cells to correctly fold and posttranslationally modify secretory proteins and transmembrane proteins (Oakes and Papa, 2015). The accumulation of unfolded proteins in the ER triggers UPR. When the UPR is persistent or excessive, ER stress triggers apoptosis (Xu et al., 2005). Increasing evidence has demonstrated that persistent and severe ERS ultimately aggravates SCIRI by activating the ERS‐related apoptosis pathway (Yamauchi et al., 2007; Mizukami et al., 2010; Zhou et al., 2016; Wu et al., 2018). BA has been reported to protect HT22 murine hippocampal neuronal cells from ER stress-induced apoptosis (Choi et al., 2010). Therefore, we hypothesized that BA downregulates ERS‐related apoptosis in SCIRI. GRP78 is a marker protein of ERS. Under SCIRI, GRP78 dissociates from transmembrane proteins, activates ERS mediated apoptotic pathways, and then induces the gene expression of CHOP and Caspase12, eventually leading to apoptosis (Ferri and Kroemer, 2001; Iurlaro and Muñoz-Pinedo, 2016). In addition, the changes in PDI activity are related to protein misfolding and ER stress (Lee et al., 2014), and ATF4 is the main regulator of the ER stress response (Jeong et al., 2019). The Western blotting results demonstrated that GRP78, PDI, ATF4, CHOP, and CASP12 expression levels were decreased in the BA group. In addition, immunofluorescence staining showed that BA decreased the percentage of CASP12-positive neurons at the spinal cord lesion. Based on these results, we concluded that BA inhibited ERS-mediated apoptosis in SCIRI.

Several studies suggested that activation of ER stress can regulate the activity of pyroptosis. Simard et al. reported that the degradation of the ER stress sensor ATF6 can lead to activation of the NLRP3 inflammasome, which further induce pyroptosis (Simard et al., 2015). Moreover, Kim et al’s findings revealed a mechanistic link that ER stress can trigger the inflammation related to pyroptosis IL-1β secretion *via* NF-κB activation (Kim et al., 2014). However, the cognition of the mechanistic relationship between pyroptosis and ER stress remains superficial and needs to be further investigation.

Autophagy is a highly conserved lysosomal pathway involving the degradation of a large number of cytoplasmic contents, characterized by the formation of double-membrane vesicles (Levine and Yuan, 2005). This pathway plays an indispensable role in cell homeostasis, energy and defense (Florey, 2018). The role of autophagy has two sides in cell survival. Autophagy can promote cell survival by removing damaged mitochondria and organelles and by degrading pathogens and macromolecular proteins in cells (Levine and Yuan, 2005), but high level of activation of autophagy may cause cell death (Uchiyama et al., 2008). However, autophagy has generally been found to be beneficial in the SCIRI model (Wang et al., 2017). Liu et al. reported that Baicalein can reduce liver ischemia/reperfusion injury by inducing HO-1-mediated autophagy (Liu et al., 2016). Therefore, we evaluated the relationship between BA and autophagy-related proteins. Our WB results demonstrated that Beclin1, ATG5, VPS34, CTSD, ATP6V1B2, and LC3II were present at higher levels in the SCIR+Baicalein group than in the Sham and SCIR+Vehicle groups, with a lower level of p62. Immunofluorescence staining (for NeuN/LC3II and NeuN/P62) also revealed that BA up-regulated the level of autophagy in neurons from the spinal cord lesions. Taken together, our results confirmed that BA inducted autophagy in SCIRI.

Autophagy, pyroptosis and ERS-mediated apoptosis are three types of programmed cell death (Fink and Cookson, 2005). Recent evidence suggests that in the process of cell death, the interaction between these cell death programs plays a fine control role in the ultimate results (Leist et al., 1997; Lockshin and Zakeri, 2004). The interactions and relationships among autophagy, pyroptosis and ERS-mediated apoptosis have been widely studied, as Li et al. have reported that autophagy protected HUVECs against ER stress-mediated apoptosis (Li et al., 2019). Increasing evidence demonstrates that hypoxic/ischemic conditions lead to mitochondrial injury (Zuo et al., 2014) and that damaged mitochondria lead to a massive accumulation of ROS (Lin et al., 2019a). Studies have clarified that the massive accumulation of ROS can induce the NLRP3 inflammasome and subsequently trigger caspase-1-dependent pyroptosis (Qiu et al., 2017). Li et al. reported that mitophagy, which can selectively remove dysfunctional mitochondria by autophagy, plays an important role in SCIRI (Li et al., 2018a). Hence, we suspect that BA facilitates the repair of spinal cord injury by inducing autophagy to dampen pyroptosis and reduce ERS-mediated apoptosis. To determine the role of autophagy in SCIRI, 3-methyladenine (3MA), a widely used autophagy inhibitor (Wang et al., 2016), was used in BA treated SCIRI mice. Our WB resulted showed that treatment with 3MA apparently decreased the expressions levels of Beclin1, ATG5, VPS34, CTSD, ATP6V1B2, and LC3II and increased the expressions levels of P62, NLRP3, GSDMD, C-CASP1, GRP78, PDI, ATF4, CHOP, and CASP12. In addition, the ELISA results showed that the levels of IL1β and IL18 expression increased in the SCIR+ Baicalein+3MA group, and the BMS results showed that compared to the SCIR+Baicalein group, the SCIR+Baicalein+3MA group had significant lower BMS scores. These results showed that 3MA reversed all the therapeutic benefits and outcomes of BA on SCIRI, demonstrating that BA-induced autophagy is responsible for inhibiting pyroptosis and ERS-mediated apoptosis.

Previous study demonstrated that the treatment of baicalein for traumatic spinal cord injury (TSCI) was promising (Li et al., 2018b). Considering the correlation between the pathological mechanisms of SCI and TSCI, baicalein may also play a crucial role in SCIRI. But no investigation about this. So our findings firstly demonstrated that baicalein exerted neuro-protection for SCIR. In addition, A large number of studies found that baicalein attenuated apoptosis *via* the augment of autophagy in various diseases (Kuang et al., 2017; Li et al., 2017; Li et al., 2018b). While few studies focused on the pharmacological effect of baicalein on other types of cell death, such necroptosis, ferroptosis, and pyroptosis. However, pyroptosis, as a new cell death type, has not been studied in SCIRI. Therefore, this is the first study to investigated the role of pyroptosis in SCIRI with baicalein treatment.

## Conclusion

**Figure 6:**
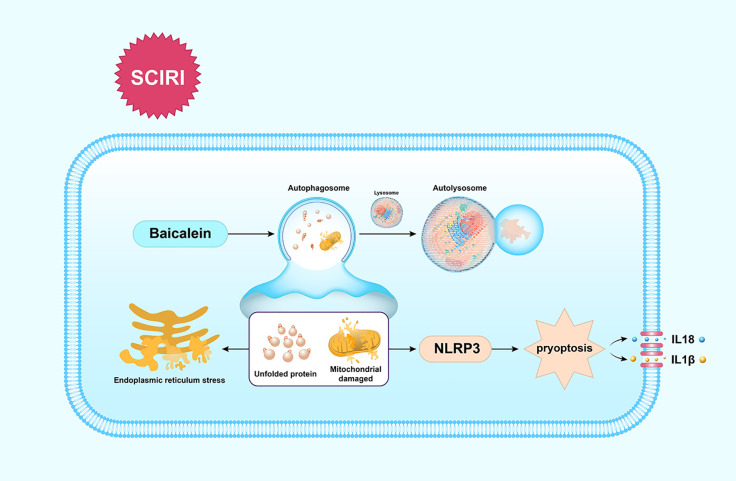
Schematic illustration of the proposed molecular mechanism by which baicalein facilitates functional recovery after spinal cord ischemia-reperfusion injury (SCIRI) by activating autophagy and subsequently reducing ER stress-induced apoptosis and attenuating pyroptosis. Spinal cord ischemia-reperfusion (I/R) injury leads to the accumulation of unfolded proteins in the endoplasmic reticulum (ER) and mitochondrial damage. Autophagy is then activated by baicalein to contribute to the elimination of damaged mitochondria and unfolded proteins by forming autolysosomes with lysosomes. Subsequently, ER stress-induced apoptosis and pyroptosis are inhibited, which further results in the promotion of functional recovery.

Our findings show that BA enhances the functional recovery of SCIRI by activating autophagy to clear unfolded protein and damaged mitochondria to reduce ERS-mediated apoptosis and dampen pyroptosis, which is summarized in **Figure 6**. These findings indicate BA maybe a promising therapeutic agent for the future treatments of SCIR.

## Data Availability Statement

The raw data supporting the conclusions of this article will be made available by the authors, without undue reservation, to any qualified researcher.

## Ethics Statement

The animal study was reviewed and approved by Animal Research Committee of Wenzhou Medical University (wydw2017-0022).

## Author Contributions

CW and HuiX wrote the manuscript text. JL, XH, XY, and YH prepared figures and collected samples. YL, SS, YW, and HuaX analyzed data. WN and KZ designed the experiment. CW, WN, and KZ revised manuscript. All authors contributed to the article and approved the submitted version.

## Funding

This work was supported by grants from Zhejiang Provincial Medicine and Health Technology Project (No. 2017KY472 to KZ; No.2015RCB022 to HuiX); Public Welfare Technology Research Project of Zhejiang Province (LGF20H150003 to KZ); Zhejiang Provincial Natural Science Foundation (No. LY17H060009 to WN); Natural Science Foundation of China (No. 81601705 to KZ, and No. 81873992 to HuaX.

## Conflict of Interest

The authors declare that the research was conducted in the absence of any commercial or financial relationships that could be construed as a potential conflict of interest.

## Abbreviations

ATF4, Recombinant Activating Transcription Factor 4; BA, Baicalein; BMS, basso mouse scale; C-CASP1, caspase 1; CTSD, cathepsin D; DAPI, 40,6-diamidino-2-phenylindole; DPI, protein disulfide isomerase; ERS, endoplasmic reticulum stress; FITC, fluorescein isothiocyanate; GAPDH, glyceraldehyde 3-phosphate dehydrogenase; GRP78, glucose-regulated protein78; LC3B, autophagy marker light chain 3B; SCIRI, Spinal cord ischemia-reperfusion injury; SQSTM1/p62, nucleoporin p62; UPR, unfolded protein response; VPS34, phosphoinositide-3-kinase–3MA, 3-methyladenine.

## References

[B1] Basso D. M. Fisher L. C. Anderson A. J. Jakeman L. B. McTigue D. M. Popovich P. G. (2006). Basso Mouse Scale for locomotion detects differences in recovery after spinal cord injury in five common mouse strains. J. Neurotrauma. 23 (5), 635–659. 10.1089/neu.2006.23.635 16689667

[B2] Byrne B. G. Dubuisson J.-F. Joshi A. D. Persson J. J. Swanson M. S. (2013). Inflammasome components coordinate autophagy and pyroptosis as macrophage responses to infection. mBio. 4 (1), e00620–12. 10.1128/mBio.00620-12 23404401 PMC3573666

[B3] Chang W.-T. Li J. Haung H.-H. Liu H. Han M. Ramachandran S. (2011). Baicalein protects against doxorubicin-induced cardiotoxicity by attenuation of mitochondrial oxidant injury and JNK activation. J. Cell. Biochem. 112 (10), 2873–2881. 10.1002/jcb.23201 21618589 PMC3178681

[B4] Chen Z. Guo H. Lu Z. Sun K. Jin Q. (2019). Hyperglycemia aggravates spinal cord injury through endoplasmic reticulum stress mediated neuronal apoptosis, gliosis and activation. Biomed. Pharmacother. 112, 108672. 10.1016/j.biopha.2019.108672 30784940

[B5] Cheng S.-B. Nakashima A. Huber W. J. Davis S. Banerjee S. Huang Z. (2019). Pyroptosis is a critical inflammatory pathway in the placenta from early onset preeclampsia and in human trophoblasts exposed to hypoxia and endoplasmic reticulum stressors. Cell Death Dis. 10 (12), 927. 10.1038/s41419-019-2162-4 31804457 PMC6895177

[B6] Choi J. H. Choi A. Y. Yoon H. Choe W. Yoon K.-S. Ha J. (2010). Baicalein protects HT22 murine hippocampal neuronal cells against endoplasmic reticulum stress-induced apoptosis through inhibition of reactive oxygen species production and CHOP induction. Exp. Mol. Med. 42 (12), 811–822. 10.3858/emm.2010.42.12.084 20959717 PMC3015155

[B7] Conrad M. F. Ye J. Y. Chung T. K. Davison J. K. Cambria R. P. (2008). Spinal cord complications after thoracic aortic surgery: long-term survival and functional status varies with deficit severity. J. Vasc. Surg. 48 (1), 47–53. 10.1016/j.jvs.2008.02.047 18486422

[B8] Dai W. Wang X. Teng H. Li C. Wang B. Wang J. (2019). Celastrol inhibits microglial pyroptosis and attenuates inflammatory reaction in acute spinal cord injury rats. Int. Immunopharmacol. 66, 215–223. 10.1016/j.intimp.2018.11.029 30472522

[B9] Faheem H. Mansour A. Elkordy A. Rashad S. Shebl M. Madi M. (2019). Neuroprotective effects of minocycline and progesterone on white matter injury after focal cerebral ischemia. J. Clin. Neurosci. 64, 206–213. 10.1016/j.jocn.2019.04.012 31023573

[B10] Ferri K. F. Kroemer G. (2001). Organelle-specific initiation of cell death pathways. Nat. Cell Biol. 3 (11), E255–E263. 10.1038/ncb1101-e255 11715037

[B11] Fink S. L. Cookson B. T. (2005). Apoptosis, pyroptosis, and necrosis: mechanistic description of dead and dying eukaryotic cells. Infect. Immun. 73 (4), 1907–1916. 10.1128/IAI.73.4.1907-1916.2005 15784530 PMC1087413

[B12] Florey O. (2018). The double life of autophagy proteins. Nat. Microbiol. 3 (12), 1334–1335. 10.1038/s41564-018-0310-8 30478385

[B13] Ha Sen Ta N. Nuo M. Meng Q.-T. Xia Z.-Y. (2019). The Pathway of Let-7a-1/2-3p and HMGB1 Mediated Dexmedetomidine Inhibiting Microglia Activation in Spinal Cord Ischemia-Reperfusion Injury Mice. J. Mol. Neurosci. 69 (1), 106–114. 10.1007/s12031-019-01338-4 31190218

[B14] Hentia C. Rizzato A. Camporesi E. Yang Z. Muntean D. M. Săndesc D. (2018). An overview of protective strategies against ischemia/reperfusion injury: The role of hyperbaric oxygen preconditioning. Brain Behav. 69 (1), 106–114. 10.1002/brb3.959 PMC5943756 29761012

[B15] Iurlaro R. Muñoz-Pinedo C. (2016). Cell death induced by endoplasmic reticulum stress. FEBS J. 283 (14), 2640–2652. 10.1111/febs.13598 26587781

[B16] Jeong M.-H. Jeong H.-J. Ahn B.-Y. Pyun J.-H. Kwon I. Cho H. (2019). PRMT1 suppresses ATF4-mediated endoplasmic reticulum response in cardiomyocytes. Cell Death Dis. 10 (12), 903. 10.1038/s41419-019-2147-3 31787756 PMC6885520

[B17] Jia X. Kowalski R. G. Sciubba D. M. Geocadin R. G. (2013). Critical care of traumatic spinal cord injury. J. Intensive Care Med. 28 (1), 12–23. 10.1177/0885066611403270 21482574

[B18] Karimipour M. Farjah G. H. Molazadeh F. Ansari M. Pourheidar B. (2019). Protective Effect of Contralateral, Ipsilateral, and Bilateral Remote Ischemic Preconditioning on Spinal Cord Ischemia Reperfusion Injury in Rats. Turk Neurosurg. 29 (6), 933–939. 10.5137/1019-5149.JTN.26237-19.3 31608967

[B19] Kim S. Joe Y. Jeong S. O. Zheng M. Back S. H. Park S. W. (2014). Endoplasmic reticulum stress is sufficient for the induction of IL-1β production via activation of the NF-κB and inflammasome pathways. Innate Immun. 20 (8), 799–815. 10.1177/1753425913508593 24217221

[B20] Kuang L. Cao X. Lu Z. (2017). Baicalein Protects against Rotenone-Induced Neurotoxicity through Induction of Autophagy. Biol. Pharm. Bull. 40 (9), 1537–1543. 10.1248/bpb.b17-00392 28659545

[B21] Lee S. Min Kim S. Dotimas J. Li L. Feener E. P. Baldus S. (2014). Thioredoxin-interacting protein regulates protein disulfide isomerases and endoplasmic reticulum stress. EMBO Mol. Med. 6 (6), 732–743. 10.15252/emmm.201302561 24843047 PMC4203352

[B22] Leist M. Single B. Castoldi A. F. Kühnle S. Nicotera P. (1997). Intracellular adenosine triphosphate (ATP) concentration: a switch in the decision between apoptosis and necrosis. J. Exp. Med. 185 (8), 1481–1486. 10.1084/jem.185.8.1481 9126928 PMC2196283

[B23] Levine B. Yuan J. (2005). Autophagy in cell death: an innocent convict? J. Clin. Invest. 115 (10), 2679–2688. 10.1172/JCI26390 16200202 PMC1236698

[B24] Li X.-Q. Lv H.-W. Tan W.-F. Fang B. Wang H. Ma H. (2014). Role of the TLR4 pathway in blood-spinal cord barrier dysfunction during the bimodal stage after ischemia/reperfusion injury in rats. J. Neuroinflamm. 11, 62. 10.1186/1742-2094-11-62 PMC3977699 24678770

[B25] Li B. Lu M. Jiang X.-X. Pan M.-X. Mao J.-W. Chen M. (2017). Inhibiting reactive oxygen species-dependent autophagy enhanced baicalein-induced apoptosis in oral squamous cell carcinoma. J. Nat. Med. 71 (12), 433–441. 10.1007/s11418-017-1076-7 28176233

[B26] Li Q. Gao S. Kang Z. Zhang M. Zhao X. Zhai Y. (2018a). Rapamycin Enhances Mitophagy and Attenuates Apoptosis After Spinal Ischemia-Reperfusion Injury. Front. Neurosci. 12, 865. 10.3389/fnins.2018.00865 30559639 PMC6286985

[B27] Li Y. Lin S. Xu C. Zhang P. Mei X. (2018b). Triggering of Autophagy by Baicalein in Response to Apoptosis after Spinal Cord Injury: Possible Involvement of the PI3K Activation. Biol. Pharm. Bull. 41 (4), 478–486. 10.1248/bpb.b17-00768 29367475

[B28] Li C.-F. Pan Y.-K. Gao Y. Shi F. Wang Y.-C. Sun X.-Q. (2019). Autophagy protects HUVECs against ER stress-mediated apoptosis under simulated microgravity. Apoptosis. 24 (9-10), 812–825. 10.1007/s10495-019-01560-w 31359205 PMC6711952

[B29] Lin D.-S. Huang Y.-W. Ho C.-S. Hung P.-L. Hsu M.-H. Wang T.-J. (2019a). Oxidative Insults and Mitochondrial DNA Mutation Promote Enhanced Autophagy and Mitophagy Compromising Cell Viability in Pluripotent Cell Model of Mitochondrial Disease. Cells. 8 (1), 65. 10.3390/cells8010065 PMC6356288 30658448

[B30] Lin Q. Li S. Jiang N. Shao X. Zhang M. Jin H. (2019b). PINK1-parkin pathway of mitophagy protects against contrast-induced acute kidney injury via decreasing mitochondrial ROS and NLRP3 inflammasome activation. Redox Biol. 26, 101254 10.1016/j.redox.2019.101254 31229841 PMC6597739

[B31] Liu A. Huang L. Fan H. Fang H. Yang Y. Liu S. (2015). Baicalein pretreatment protects against liver ischemia/reperfusion injury via inhibition of NF-κB pathway in mice. Int. Immunopharmacol. 24 (1), 72–79. 10.1016/j.intimp.2014.11.014 25479717

[B32] Liu A. Huang L. Guo E. Li R. Yang J. Li A. (2016). Baicalein pretreatment reduces liver ischemia/reperfusion injury via induction of autophagy in rats. Sci. Rep. 6, 25042. 10.1038/srep25042 27150843 PMC4858649

[B33] Lockshin R. A. Zakeri Z. (2004). Apoptosis, autophagy, and more. Int. J. Biochem. Cell Biol. 36 (12), 2405–2419. 10.1016/j.biocel.2004.04.011 15325581

[B34] Miranda V. Sousa J. Mansilha A. (2018). Spinal cord injury in endovascular thoracoabdominal aortic aneurysm repair: prevalence, risk factors and preventive strategies. Int. Angiol. 37 (2), 112–126. 10.23736/S0392-9590.18.03960-3 29424186

[B35] Mizukami T. Orihashi K. Herlambang B. Takahashi S. Hamaishi M. Okada K. (2010). Sodium 4-phenylbutyrate protects against spinal cord ischemia by inhibition of endoplasmic reticulum stress. J. Vasc. Surg. 52 (6), 1580–1586. 10.1016/j.jvs.2010.06.172 20843623

[B36] Oakes S. A. Papa F. R. (2015). The role of endoplasmic reticulum stress in human pathology. Annu. Rev. Pathol. 10, 173–194. 10.1146/annurev-pathol-012513-104649 25387057 PMC5568783

[B37] Qiu Z. Lei S. Zhao B. Wu Y. Su W. Liu M. (2017). NLRP3 Inflammasome Activation-Mediated Pyroptosis Aggravates Myocardial Ischemia/Reperfusion Injury in Diabetic Rats. Oxid. Med. Cell Longev. 2017, 9743280. 10.1155/2017/9743280 29062465 PMC5618779

[B38] Qiu Z. He Y. Ming H. Lei S. Leng Y. Xia Z.-Y. (2019). Lipopolysaccharide (LPS) Aggravates High Glucose- and Hypoxia/Reoxygenation-Induced Injury through Activating ROS-Dependent NLRP3 Inflammasome-Mediated Pyroptosis in H9C2 Cardiomyocytes. J. Diabetes Res. 2019, 8151836. 10.1155/2019/8151836 30911553 PMC6398034

[B39] Rathinam V. A. K. Vanaja S. K. Fitzgerald K. A. (2012). Regulation of inflammasome signaling. Nat. Immunol. 13 (4), 333–342. 10.1038/ni.2237 22430786 PMC3523703

[B40] Shi J. Gao W. Shao F. (2017). Pyroptosis: Gasdermin-Mediated Programmed Necrotic Cell Death. Trends Biochem. Sci. 42 (4), 245–254. 10.1016/j.tibs.2016.10.004 27932073

[B41] Shi H. Zhang Y. Xing J. Liu L. Qiao F. Li J. (2020). Baicalin attenuates hepatic injury in non-alcoholic steatohepatitis cell model by suppressing inflammasome-dependent GSDMD-mediated cell pyroptosis. Int. Immunopharmacol. 81, 106195 10.1016/j.intimp.2020.106195 32028242

[B42] Simard J.-C. Vallieres F. de Liz R. Lavastre V. Girard D. (2015). Silver nanoparticles induce degradation of the endoplasmic reticulum stress sensor activating transcription factor-6 leading to activation of the NLRP-3 inflammasome. J. Biol. Chem. 290 (9), 5926–5939. 10.1074/jbc.M114.610899 25593314 PMC4342498

[B43] Uchiyama Y. Koike M. Shibata M. (2008). Autophagic neuron death in neonatal brain ischemia/hypoxia. Autophagy. 4 (4), 404–408. 10.4161/auto.5598 18212531

[B44] Wang X. Zhou G. Liu C. Wei R. Zhu S. Xu Y. (2016). versusAcanthopanax 3-Methyladenine Ameliorates Sodium Taurocholate-Induced Severe Acute Pancreatitis by Inhibiting the Autophagic Pathway in Rats. Mediators Inflamm. 2016, 8369704 10.1155/2016/8369704 28115794 PMC5225378

[B45] Wang L. Feng D. Liu Y. Li S. Jiang L. Long Z. (2017). Autophagy plays a protective role in motor neuron degeneration following spinal cord ischemia/reperfusion-induced spastic paralysis. Am. J. Transl. Res. 9 (9), 4261–4270. 28979699 PMC5622268

[B46] Wu H. Huang T. Ying L. Han C. Li D. Xu Y. (2016). MiR-155 is Involved in Renal Ischemia-Reperfusion Injury via Direct Targeting of FoxO3a and Regulating Renal Tubular Cell Pyroptosis. Cell. Physiol. Biochem. 40 (6), 1692–1705. 10.1159/000453218 28006785

[B47] Wu F. Wei X. Wu Y. Kong X. Hu A. Tong S. (2018). Chloroquine Promotes the Recovery of Acute Spinal Cord Injury by Inhibiting Autophagy-Associated Inflammation and Endoplasmic Reticulum Stress. J. Neurotrauma. 35 (12), 1329–1344. 10.1089/neu.2017.5414 29316847

[B48] Xia P. Pan Y. Zhang F. Wang N. Wang E. Guo Q. (2018). Pioglitazone Confers Neuroprotection Against Ischemia-Induced Pyroptosis due to its Inhibitory Effects on HMGB-1/RAGE and Rac1/ROS Pathway by Activating PPAR-γ. Cell. Physiol. Biochem. 45 (6), 2351–2368. 10.1159/000488183 29554649

[B49] Xing S.-S. Yang J. Li W.-J. Li J. Chen L. Yang Y.-T. (2020). Salidroside Decreases Atherosclerosis Plaque Formation via Inhibiting Endothelial Cell Pyroptosis. Inflammation. 43 (2), 433–440. 10.1007/s10753-019-01106-x 32076940

[B50] Xu C. Bailly-Maitre B. Reed J. C. (2005). Endoplasmic reticulum stress: cell life and death decisions. J. Clin. Invest. 115 (10), 2656–2664. 10.1172/JCI26373 16200199 PMC1236697

[B51] Yamauchi T. Sakurai M. Abe K. Matsumiya G. Sawa Y. (2007). Impact of the endoplasmic reticulum stress response in spinal cord after transient ischemia. Brain Res. 1169, 24–33. 10.1016/j.brainres.2007.06.093 17707355

[B52] Zhang Z. Cui W. Li G. Yuan S. Xu D. Hoi M. P. M. (2012). Baicalein protects against 6-OHDA-induced neurotoxicity through activation of Keap1/Nrf2/HO-1 and involving PKCα and PI3K/AKT signaling pathways. J. Agric. Food Chem. 60 (33), 8171–8182. 10.1021/jf301511m 22838648

[B53] Zhang L. Liu H. Jia L. Lyu J. Sun Y. Yu H. (2019). Exosomes Mediate Hippocampal and Cortical Neuronal Injury Induced by Hepatic Ischemia-Reperfusion Injury through Activating Pyroptosis in Rats. Oxid. Med. Cell Longev. 2019, 3753485. 10.1155/2019/3753485 31814872 PMC6878784

[B54] Zhao L. Zhai M. Yang X. Guo H. Cao Y. Wang D. (2019). Dexmedetomidine attenuates neuronal injury after spinal cord ischaemia-reperfusion injury by targeting the CNPY2-endoplasmic reticulum stress signalling. J. Cell. Mol. Med. 23 (12), 8173–8183. 10.1111/jcmm.14688 31625681 PMC6850922

[B55] Zheng Z. Li G. (2020). Mechanisms and Therapeutic Regulation of Pyroptosis in Inflammatory Diseases and Cancer. Int. J. Mol. Sci. 21 (4), 1456. 10.3390/ijms21041456 PMC7073143 32093389

[B56] Zhou Y. Ye L. Zheng B. Zhu S. Shi H. Zhang H. (2016). Phenylbutyrate prevents disruption of blood-spinal cord barrier by inhibiting endoplasmic reticulum stress after spinal cord injury. Am. J. Transl. Res. 8 (4), 1864–1875. 27186310 PMC4859915

[B57] Zuo W. Zhang S. Xia C.-Y. Guo X.-F. He W.-B. Chen N.-H. (2014). Mitochondria autophagy is induced after hypoxic/ischemic stress in a Drp1 dependent manner: the role of inhibition of Drp1 in ischemic brain damage. Neuropharmacology. 86, 103–115. 10.1016/j.neuropharm.2014.07.002 25018043

